# CpG oligodeoxynucleotide CpG-685 upregulates functional interleukin-21 receptor on chronic lymphocytic leukemia B cells through an NF-κB mediated pathway

**DOI:** 10.18632/oncotarget.3285

**Published:** 2015-03-26

**Authors:** Rebekah L. Browning, Xiaokui Mo, Natarajan Muthusamy, John C. Byrd

**Affiliations:** ^1^ Division of Hematology, Department of Internal Medicine, College of Medicine, The Ohio State University, Columbus, Ohio, United States of America; ^2^ Center for Biostatistics, The Ohio State University, Columbus, Ohio, United States of America

**Keywords:** chronic lymphocytic leukemia, interleukin-21, CpG ODN, NF-κB

## Abstract

CpG oligodeoxynucleotides (ODNs) upregulate the interleukin-21 receptor (IL21R) and enhance IL-21-mediated cytotoxicity in chronic lymphocytic leukemia (CLL) B cells. We demonstrate that treatment of CLL B cells with the ODN CpG-685 leads to increased IL21R expression, and that this increased expression enhances the effects of IL-21 treatment as evidenced by increased phosphorylation of JAK1, STAT1, and STAT3, as compared to IL-21 treatment without prior CpG stimulation. Induction of IL21R by CpG-685 also enhanced IL-21-mediated cytotoxicity. The mechanism by which CpG ODNs upregulate IL21R has not been elucidated, although IL21R regulation in T cells has been shown to be linked to T cell receptor-induced Sp1 binding to the IL21R promoter. Here, we demonstrate that luciferase reporter constructs containing the Sp1 binding site have increased basal luciferase activity compared to constructs lacking the Sp1 binding site, but fail to increase luciferase activity with CpG-685 stimulation in CLL B cells. By treating CLL cells with an NF-κB inhibitor, we inhibit the CpG ODN-mediated induction of IL21R, thus demonstrating that CpG-685 upregulates IL21R through an NF-κB mediated pathway. These findings suggest an alternative mechanism for induction of IL-21 receptor in CLL B cells and provide a basis for creation of future combination therapies.

## INTRODUCTION

Chronic lymphocytic leukemia (CLL) is the most prevalent adult leukemia and is characterized by an accumulation of malignant CD5+, CD19+, CD20+, CD23+ B cells that exert immunosuppressive activity. Thus, CLL patients often experience significant immune dysfunction; consequently, infection is a major cause of mortality in CLL patients [1, 2]. Exacerbating this problem, most currently approved therapies for CLL are immunosuppressive to either the T-cell or B-cell adaptive immune system, further impairing immune function. In addition, CLL is incurable with the exception of stem cell transplant, which is not an option for the majority of CLL patients. Thus there is an urgent need for new therapeutic strategies that can target the tumor cell population while enhancing activity of other immune cells. Interleukin-21 (IL-21) has emerged as a potential treatment of this nature.

IL-21 is a member of the IL-2 family of cytokines and signals through its receptor IL21R, which functions as a heterodimer with the common gamma chain [3, 4]. IL-21 is produced by activated CD4+ T cells and has pleiotropic effects on B, T, and NK cells [5–7]. IL-21 has been shown to have cytotoxic effects on CLL cells when used as a single agent, including in CLL cells activated by CD40L or CpG oligodeoxynucleotides (ODN) [8–10]. However, the expression of IL21R varies greatly between patients [9]. This variability is of particular interest because receptor expression is directly correlated with the apoptotic action of IL-21 in CLL cells [8].

Both CpG ODNs and CD40L have been shown to upregulate IL21R in CLL cells and enhance IL-21-mediated killing [9, 10]. However, the mechanism by which these agents upregulate IL21R is unknown. In T cells, regulation of IL21R has been demonstrated to be subject to T cell receptor-mediated Sp1 transcription factor activity [11]. As CpG ODNs are currently in development for use as an immunomodulatory agent in CLL, it becomes important to understand the mechanism by which IL21R is regulated in this disease. Here, we show that while Sp1 binding may be involved in baseline expression of IL21R in CLL cells, the upregulation of IL21R by CpG ODNs is mediated through NF-κB.

## RESULTS

### CPG-685 upregulates functionally competent IL21R

**Figure 1:**
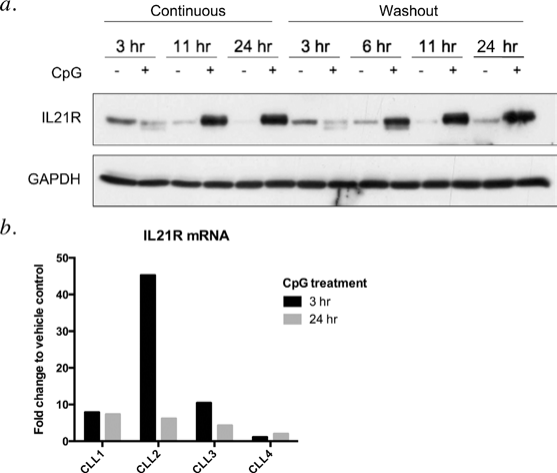
CpG-685 treatment of CLL B cells enhances expression of IL21R in a time-dependent manner **a.** Immunoblot analysis of lysates from CLL patient cells exposed to CpG-685 (1.7 μM) for 3 hours, then incubated in fresh media for 0–21 additional hours (times shown are total incubation times). Blot was reprobed with anti-GAPDH antibody to show equal loading. Representative blot, *n* = 5. **b.** Real-time RT-PCR analysis of CLL cells incubated with CpG-685 for 3 or 24 hours. Raw values were normalized to 18s internal control transcript and are shown as fold change relative to time-matched untreated controls.

CpG ODNs have been reported to trigger apoptosis in CLL cells, with CpG-685 showing more potent activity than CpG 2006 [12]. CpG 2006 has been shown to upregulate IL21R in B CLL cells, leading to enhanced IL-21-mediated apoptosis of the tumor cells [10]. CpG-685, also a Class B CpG ODN [13, reviewed in 14] has not been examined in the setting of combination therapy with IL-21. To assess whether CpG-685 upregulates the IL21R on CLL cells, lysates from CpG-685-treated CLL patient cells were probed for IL21R protein. Treatment of CLL cells with CpG-685 led to a time-dependent increase in IL21R, as compared to untreated controls (Figure 1a). Although some patient samples showed an initial decrease at three hours of treatment, levels recovered and exceeded baseline levels within six hours after addition of CpG ODN, with maximum levels achieved between six and eleven hours of stimulation. In order to better approximate physiological conditions, additional experiments were done in which the CpG ODN was washed out of culture after three hours of treatment. This transient exposure was sufficient to induce IL21R, as IL21R levels still increased at later time points when CLL cells were exposed to CpG-685 for just 3 hours, followed by washout and incubation in CpG-free media (Figure 1a). Quantitative RT-PCR analysis indicated that CpG-685-mediated upregulation of IL21R occurs at the transcriptional level, with increased IL21R mRNA occurring within 3 hours of CpG-685 stimulation (Figure 1b).

**Figure 2:**
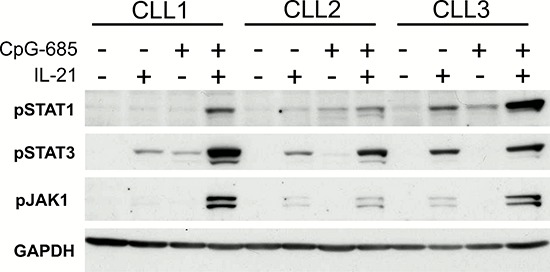
CpG-induced IL21R demonstrates functional signaling **a.** Immunoblot analysis of lysates from CLL B cells treated with CpG-685 for 3 hours, washed, then incubated in fresh media for a total time of 24 hours, followed by 15 minutes of treatment with IL21. 5/8 patients showed increased pSTAT1 with CpG+IL21 compared to IL21 alone, 7/8 patients showed increased pSTAT3, and 7/8 showed increase pJAK1. Image shows results from three representative patient samples. Blot was reprobed with anti-GAPDH antibody to show equal loading.

Having confirmed that CpG-685 was capable of inducing IL21R on CLL cells, we next assessed whether the upregulated IL21R was functionally competent. CLL cells from eight patients were treated with CpG-685 for three hours, washed, incubated in fresh media for 24 hours, then stimulated with IL-21 for 15 minutes. Lysates were assessed for phosphorylation of the IL-21 downstream targets STAT1, STAT3, and JAK1. These same patient samples were also assessed for induction of IL21R, in order to confirm that each was responsive to CpG-685 treatment. As shown in Figure 2, CpG-685-treated samples showed an increase in pSTAT1^Y701^, pSTAT3^Y705^, and pJAK1^Y1022/Y1023^ in response to IL-21 stimulation, as compared to IL-21 treatment only. These results indicate that the IL21R induced by CpG-685 is indeed functional in primary CLL cells.

**Figure 3:**
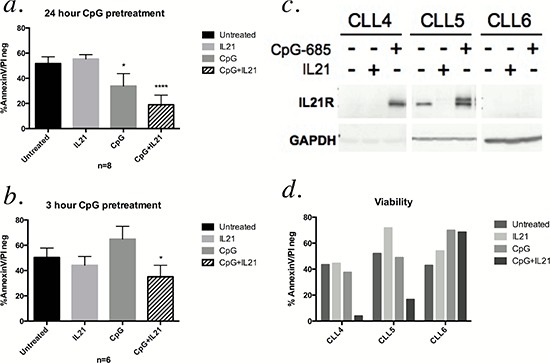
Pretreatment with CpG-685 enhances IL-21-mediated cytotoxicity **a.** AnnexinV-FITC/PI flow cytometry analysis of CLL cells treated for 24 hours with CpG-685 followed by 72 hours of IL-21 treatment. Viability graphs show percentage of cells negative for both AnnexinV-FITC and PI (* for *p* < .05, **** for *p* < .0001, compared with untreated). Data are represented as mean +/− SEM. Individual values by patient are shown in Supplemental Figure 1a. **b.** AnnexinV-FITC/PI analysis of CLL cells treated with CpG-685 for three hours, followed by washout and incubation in fresh media for eight hours prior to addition of IL-21. Cells were incubated with IL-21 for 72 hours. Individual values by patient are shown in Supplemental Figure 1b. **c and d.** Immunoblot assessment of IL21R induction (c) and viability as assessed with AnnexinV-FITC/PI flow (d) for three patient samples that failed to show cytotoxicity with IL-21 or CpG-685 alone.

Both IL-21 and CpG ODNs have cytotoxic activity in CLL cells [8–10, 12, 15–17], and the combination of IL-21 and CpG 2006 has been shown to be synergistic in inducing apoptosis of B CLL cells [10, 17]. To assess if similar findings were observed with CpG-685, we assessed apoptosis of CLL patient cells following incubation with IL-21, CpG-685, or the two combined. As shown in Figure 3a, CpG-685 treatment significantly enhanced IL-21 mediated cytotoxicity over that observed with IL-21 alone (Figure 3a, Figure S1a). As initial viability studies were done using a24-hour pretreatment with CpG, we repeated the studies using three-hour CpG exposures, followed by washout prior to addition of IL-21 for a 72-hour incubation. CpG as a single agent failed to show cytotoxic effects with a 3-hour exposure, but the combination of CpG-685 and IL-21 significantly reduced viability as compared to the untreated controls (Figure 3b, Figure S1b).

Interestingly, in a small subset of patient samples that failed to exhibit reduced viability in response to treatment with single agent IL-21 or CpG-685, two patient samples that showed an upregulation of IL21R following treatment with CpG-685 also showed apoptosis when IL-21and CpG-685 were used in combination. The single patient sample that failed to upregulate IL21R in response to CPG-685 treatment also failed to respond to combined IL-21and CPG-685 (Figure 3c and 3d). This patient sample also showed a decrease of Toll-Like Receptor 9 (TLR9) protein over time (Supplementary Figure S2), which could account for the failure of this sample to upregulate IL21R, as CpG ODNs signal through TLR9 [18]. Collectively, these data suggest that the induction of IL21R by CpG-685 may play a role in the potential therapeutic effects of combined CpG ODN and IL-21 as a novel immune therapeutic approach for CLL.

### CpG-685-mediated IL21R induction occurs via an Sp1-independent mechanism

**Figure 4:**
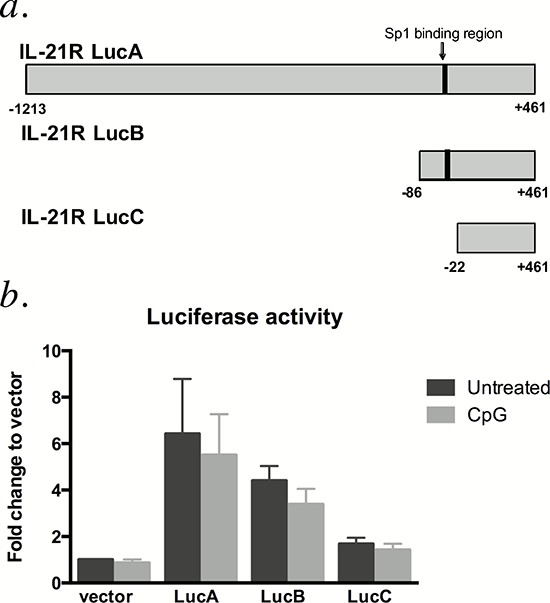
IL21R promoter constructs containing the Sp1 binding site are not responsive to CpG-685 treatment **a.** IL21R promoter luciferase reporter constructs were designed containing the Sp1 binding site (−49 to −44 bp relative to the transcription initiation site **b.** Luciferase activity in CLL B cells transiently transfected with the reporter constructs and left untreated or treated with CpG-685. All luciferase values are normalized to control renilla luciferase activity and then normalized to untreated empty vector control. Data are represented as mean +/− SEM.

Previous work indicates that the transcriptional induction of IL21R in T cells is mediated by T cell receptor-induced Sp1 transcription factor binding [11]. To assess the importance of Sp1 activity in regulation of IL21R in CLL cells, luciferase reporter constructs containing the Sp1 binding site of the IL21R promoter (the TGGGCG motif −49 to −44 bp from the major transcription initiation site [11], Figure 4a) were transiently transfected into CLL patient cells. The transfected cells were briefly allowed to recover, then incubated with or without CpG-685. As shown in Figure 4b, reporter constructs containing the Sp-1 binding site produced higher luciferase activity compared to those without the Sp1 site, consistent with prior studies in T-cells showing that this transcription factor is important in IL21R expression. However, in all four patient samples tested, CpG-685 failed to increase luciferase activity relative to the untreated controls. This result indicates that while Sp1 may play a role in basal expression of IL21R, CpG-685-mediated induction of IL21R is not dependent on this Sp1 binding site.

### IKK-β inhibition blocks CpG-685-mediated upregulation of IL21R

**Figure 5:**
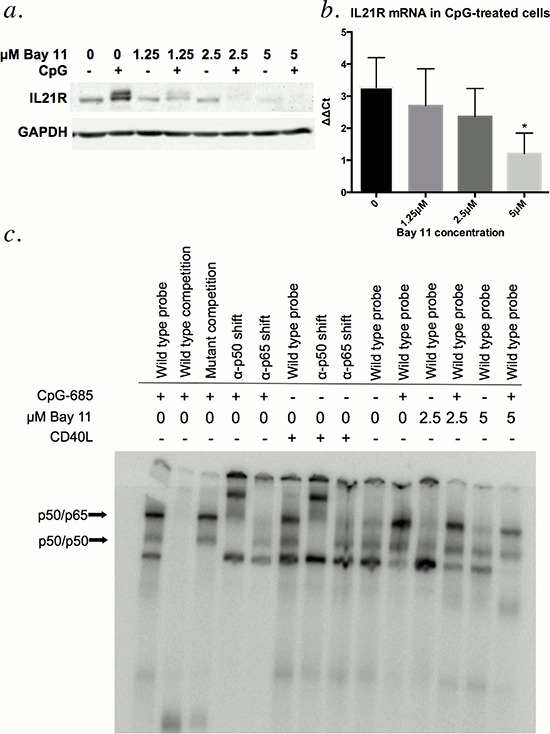
Treatment with CLL B cells with Bay 11 diminishes CpG-685-mediated upregulation of IL21R **a and b.** Evaluation of upregulation of IL21R protein by immunoblot (a) and IL21R mRNA by real time RT-PCR (b) in CLL cells treated with increasing concentrations of NF-κB inhibitor Bay 11 prior to addition of CpG-685 (3 hour exposure to CpG-685, wash, and media incubation for 8 additional hours). Real time RT-PCR data is shown as ΔΔCt = vehicle control(IL21R-18S)-CpG(IL21R-18S). Data are represented as mean +/− SEM. *n* = 5. Individual results by patient are shown in Supplemental Figure 3. Data from a sixth patient were excluded as an influential outlier (S3). **c.** Electrophoretic mobility shift assay assessment of binding of NF-κB consensus probe to nuclear extracts from CLL cells treated with 2.5 μM or 5 μM Bay 11 followed by CpG-685. Anti-p50 and anti-p65 antibodies were included for supershift. Wild type cold probe and mutant cold probe competition were included to show specificity of the interaction. CD40 ligand was used as a positive control for Nf-κB activation.

Given that both CpG ODN and CD40L enhance NF-κB binding [reviewed in 18 and 19] and promote IL21R expression in CLL cells [9, 16], we hypothesized that NF-κB was involved in upregulation of IL21R by CpG-685. To determine if inhibition of NF-κB activity would in turn suppress CpG-685-mediated upregulation of IL21R, CLL patient cells were incubated concurrently with CpG-685 and the IKK-β inhibitor Bay 11. Bay 11 treatment inhibited CpG-685-mediated induction of IL21R protein (Figure 5a) and mRNA (Figure 5b, Figure S3) in CLL cells in a dose-dependent manner. To confirm that Bay 11 indeed blocked NF-κB activation under these conditions, electrophoretic mobility shift assays were performed using a probe containing a consensus NF-κB binding site and nuclear extracts from Bay 11 and CpG-treated CLL cells. Antibodies to NF-κB p50 and p65 were included to identify the relevant complexes by supershift. As shown in Figure 5c, the addition of Bay 11 blocks both basal and CpG-685-induced binding of NF-κB complexes with the labeled probe. Together, these studies show that NF-κB is a crucial mediator of IL21R upregulation by CpG-685 in CLL B cells.

## DISCUSSION

IL-21 presents a potential immunomodulatory therapy with cytotoxic activity in CLL B cells [8–10]. Its activity, however, is dependent upon the expression of IL21R on the tumor cells [8]. CpG ODNs have been demonstrated to upregulate the receptor and enhance the activity of IL-21 [10, 17]. In addition, CpG ODNs by themselves have cytotoxic activity in CLL cells *in vitro* [12, 15, 16] and are being developed as a therapeutic approach with immune-stimulating potential [reviewed in 20 and 21]. In a phase I clinical trial of single-dose CpG 7909 (CpG 2006) in patients with relapsed CLL, patients who received IV CpG 7909 showed enhanced CD20, CD86, and TRAIL expression on CLL B cells, as well as increased NK and T cell counts [22], highlighting the potential of CpG oligodeoxynucleotides to be used as an immune-activating agent in combination therapies. Because CpG 2006 has been reported to contribute to chemoresistance to bendamustine and fludarabine in CLL cells when combined with CD40 ligation in bone marrow stromal coculture experiments [23], CpG ODNs may be best utilized in combination with immune-based therapies that could benefit from the enhanced costimuatory molecule expression on the CLL cells, along with enhanced activation of other immune cells, rather than chemotherapy based treatments. Therefore, IL-21 and CpG ODNs present a potential therapeutic combination for the treatment of CLL. Importantly, this therapy would differ from others used in CLL in that it lacks immune suppressive activity.

There are multiple potential mechanisms by which the combination of CpG stimulation followed by IL-21 treatment leads to enhanced cytotoxicity in CLL B cells. One potential reason for increased apoptosis may be increased STAT1 phosphorylation. Liang et al. [12] demonstrated that inhibition of NF-κB and the JAK/STAT inhibited CpG-induced apoptosis in CLL cells, indicating that both of these pathways are involved. They speculated that CpG treatment might induce cytokine production that could contribute to apoptosis via autocrine stimulation. Jahrsdorfer et al. [10] demonstrated that IL-21 induced granzyme B production in CLL cells, and this granzyme B was capable of inducing cytotoxicity in surrounding CLL B cells. Furthermore, production of granzyme B was increased by combining IL-21 with CpG ODN stimulation [10]. In addition, we observed apoptosis in response to combined CpG and IL-21 in CLL patient cells that failed to respond to either agent in isolation. Therefore, there is strong rationale for use of Class B CpGs and IL-21 in tandem.

In contrast to what was observed by Liang et al. [12] using CpG 2006, we did not see a marked increase in STAT1 phosphorylation with CpG-685 treatment alone. This is likely due to differences in experimental conditions; although both studies evaluated STAT1 phosphorylation at the 24-hour time point, our treatment involved washing out the CpG-685 after three hours of incubation. This suggests that sustained exposure is required for STAT1 phosphorylation *in vitro*. Nevertheless, a three-hour exposure time was sufficient to enhance IL-21-mediated phosphorylation of STAT1 at the 24-hour time point. Hagn et al. [17] did not observe a similar increase in IL-21-mediated STAT1 phosphorylation when combined with CpG ODN, and indeed showed no phosphorylation of JAK1. This difference is most likely due to the concurrent addition of CpG ODN and IL-21, rather than pre-treating the cells with CpG ODN prior to addition of IL-21. Here we show that incubating the cells with CpG-685 for three hours (followed by an additional 21-hour period without CpG-685) allowed for induction of IL-21 receptor prior to addition of the cytokine; this may account for the enhanced STAT phosphorylation observed in our data.

In addition to triggering apoptosis, treatment of CLL cells with Class B CpG ODNs enhances their potential immunogenicity to T cells by increasing costimulatory surface markers [12, and our own unpublished observations]. IL-21 promotes development of cytotoxic T cells [24] and suppresses T regulatory cell formation [25] thereby enhancing T cell responses. In this context, the combination of IL-21 and CpG-685 has potential not only to directly target CLL B cells but also to enhance the patient's immune response, recruiting other immune cells to target the tumor.

Although previous reports show that CpG ODNs and IL-21 produce superior anti-CLL cell activity when combined *in vitro*, the mechanism by which CpG ODNs induce IL21R and thereby enhance its effects has hitherto not been elucidated. Sp1 binding has been shown to a critical regulator of IL21R expression in T cells [11]; however, in CLL cells, our luciferase assays with the promoter containing the Sp1 binding site failed to show increased activity with CpG stimulation, although it does appear that Sp1 binding may play a role in endogenous expression of IL21R. Through inhibitor studies, we have demonstrated that the CpG ODN-induced IL21R expression is dependent on activation of NF-κB. Although the NF-κB pathway is generally understood to contribute to the survival of CLL cells [reviewed in 26], Liang et al. [12] demonstrated that it was necessary for the CpG ODN-induced apoptosis in CLL cells. Variable activation of the NF-κB pathway could account for the high interpatient variability of IL21R expression that was reported by de Totero et al [9] and could also explain the induction of IL21R in CLL cells following CD40 ligation. This could also account for the isolated CLL patient sample that showed decreasing TLR9 and failure to upregulate IL21R with CpG-685 stimulation, as NF-κB activation by CpG ODNs is mediated through the TLR9 signaling pathway. These findings demonstrate the mechanism for induction of IL-21 receptor in CLL B cells and provide a basis for the design of combination therapies impacting this target.

## MATERIALS AND METHODS

### Cell isolation and culture

The Ohio State University Institutional Review Board reviewed and approved the protocol for acquisition of CLL samples used in these laboratory studies. All patients gave written informed consent prior to any sample being collected. All patients had CLL as defined by the IWCLL 2008 criteria [27]. CLL B cells were selected using Rosette-Sep reagent (Stem Cell Technologies, Vancouver, BC, Canada) and isolated by Ficoll density gradient centrifugation (Ficoll-Paque Plus, Amershan Biosciences, Piscataway, NJ). Cells were suspended in RPMI 1640 medium (Gibco, Carlsbad, CA) supplemented with 10% heat-inactivated human serum, 2 mmol/l L-glutamine (Invitrogen, Carlsbad, CA), and 100 U/ml penicillin/100 μg/ml streptomycin (Sigma-Aldrich, St. Louis, MO). Cells were incubated at 37°C in an atmosphere of 5% CO_2_.

### Antibodies and Cytokines/Reagents

The ODN CpG-685 (5′-TCGTCGACGTCGTTCGTTCTC-3′, also known as GNKG168) oligodeoxynucleotide was provided by SBI Biotech Co. (Tokyo, Japan) or purchased from Sigma-Aldrich and was used at 1.7 μM. Recombinant human Il-21 was kindly provided by ZymoGenetics (Seattle, WA) and was used at 100 ng/ml. The NF-κB inhibitor Bay 11 was purchased from Calbiochem (San Diego, CA) and was used at 1.25, 2.5, and 5 μM. Rabbit anti-human phospho-STAT1^Y701^ (D4A7), mouse anti-human total STAT1 (9H2), rabbit anti-human phospho-STAT3^Y705^ (#9131), and mouse anti-human total STAT3 (124H6) were purchased from Cell Signaling Technology (Beverly, MA). Goat anti-human IL21R (L-20) and mouse anti-human GAPDH (0411) were purchased from Santa Cruz Biotechnology (Santa Cruz, CA). Rabbit anti-human p-JAK1^Y1022/Y1023^ was purchased from BioSource International (Camarillo, CA).

### Immunoblots

Whole cell lysates were prepared from 3–4 × 10^7^ cells and stored at −80°C. Protein concentration was quantified using the bicinchoninic acid assay (Pierce, Rockford, IL). Lysates were separated on 10% sodium dodecyl sulfate-polyacrylamide gels, transferred to nitrocellulose membranes (Bio-Rad Laboratories, Richmond, CA), and blocked in 5% nonfat milk prior to incubation with primary and secondary antibodies. Protein-antibody complexes were detected using SuperSignal chemiluminescent substrate (Pierce). Quantitation was performed using the Fluorchem (Alpha Innotech, San Leandro, CA). Target proteins were normalized to GAPDH values for each sample.

### RNA extraction, reverse transcription, and real time PCR

Cells were lysed in Trizol reagent (Invitrogen, Life Technologies, Grand Island, NY) and RNA was extracted according to the manufacturer's instructions. Reverse transcription was performed using the Superscript First Strand System (Invitrogen). Real time polymerase chain reaction was performed using the IL21R (Hs00222310_m1) Taqman Gene Expression Assay (Applied Biosystems, Foster City, CA). Transcript levels were determined from threshold cycle numbers, normalized to internal control 18S, and expressed relative to transcripts in untreated or vehicle-treated CLL cells.

### Assessment of apoptosis by flow cytometry

The apoptosis of cells following treatment with CPG-685 and/or IL-21 was measured using Annexin V-FITC/propidium iodide (PI) flow cytometry using a Beckman Coulter FC500 cytometer (Beckman Coulter, Indianapolis, IN). Cells were stained with Annexin V-FITC and PI according to the manufacturer's protocol (BD Pharmingen, San Diego, CA). Unstained cells and cells stained with Annexin V-FITC or PI only were also processed for compensation. Viability results are presented as percentage of cells that were negative for both Annexin V-FITC and PI.

### IL21R promoter luciferase assay

The 5′ regulatory region of the IL21R gene from −1441, −314, or −250 to +233 was subcloned in the pGL3-Basic luciferase reporter vector that had been modified to contain AscI and EcoRV restriction sites. Plasmid DNA was prepared with Qiagen Plasmid Mini and Maxi kits (Valencia, CA). Primary CLL B cells were transfected using the Amaxa nucleofector system (Lonza, Walkersville, MD) with 5 μg of the reporter construct plasmid and 1 μg of renilla luciferase vector as a transfection control. Following transfection, cells were rested briefly then were treated with CPG-685 as mentioned above. Luciferase activity was assessed using the Promega Dual Luciferase Assay (Madison, WI).

### Electrophoretic mobility shift assay (EMSA)

Nuclear extracts were prepared from CLL cells using the NE-PER kit (Pierce). NF-κB consensus (5′-AGT TGA GGG GAC TTT CCC AGG C-3′) and mutant (5′-AGT TGA GGC GAC TTT CCC AGG C-3′) probes were purchased from Santa Cruz Biotechnology and ^32^P-labeled using the Nick Translation System (Invitrogen). Five μg nuclear protein was incubated at room temperature for 30 minutes in binding buffer (10 mmol/L Tris-HCl, pH 7.5; 1.0 mmol/L ethylenediaminetetraacetic acid (EDTA); 4% Ficoll; 1.0 mmol/L dithiothreitol; 75 mmol/L KCl) plus 250 ng poly dI-dC (Sigma). Protein/probe complexes were separated on non-denaturing polyacrylamide gel in Tris borate EDTA buffer (89 mM Tris-base, 89 mM boric acid, 2.0 mM EDTA), dried, and autoradiographed. For antibody shift experiments, antibodies to NF-κB subunits p65 or p50 (Santa Cruz Biotechnology) were incubated with nuclear extract for 10 minutes prior to the addition of probe.

### Statistics

Data analyses were conducted using SAS 9.3 (SAS, Inc., Cary, NC). For the experiments with repeated measures, data were analyzed by mixed effect models incorporating observational dependencies. For the experiments involving independent groups, data were analyzed by analysis of variance. Multiplicities were adjusted by Holm's method to control the family-wise error rate at 0.05.

## SUPPLEMENTARY FIGURES


