# Supplementary material for: CpG oligodeoxynucleotide CpG-685 upregulates functional interleukin-21 receptor on chronic lymphocytic leukemia B cells through an NF-κB mediated pathway
Source: Oncotarget. 2015 Mar 26;6(18):15931–9. doi: 10.18632/oncotarget.3285 (PMC4599247; doi:10.18632/oncotarget.3285)
Supplement: Supplementary file 1 [file oncotarget-06-15931-s001.pdf]

SUPPLEMENTARY FIGURES

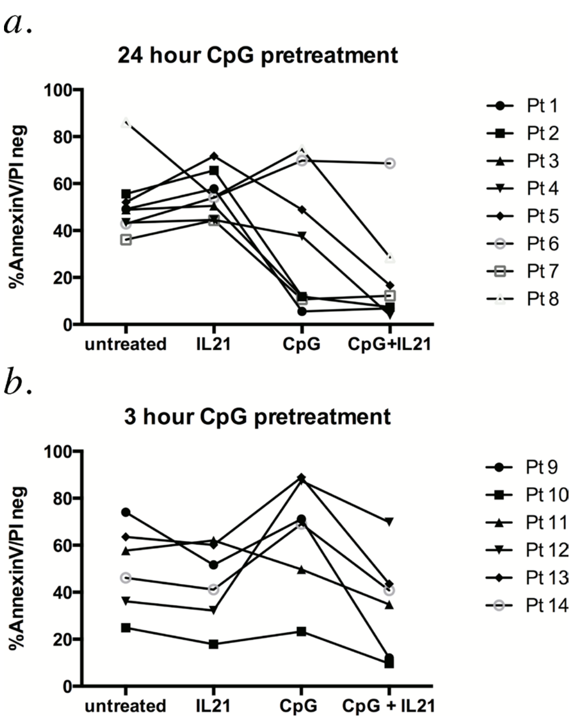

Supplementary Figure S1: Pretreatment with CpG-685 enhances IL-21-mediated cytotoxicity. a. 24 hour treatment with CpG-685 followed by 72 hours of IL-21 induces apoptosis in CLL B cells. **b.** 3 hour treatment with CpG followed by washout and 8 hour rest prior to 72 hour treatment with IL-21 induces apoptosis in CLL B cells.

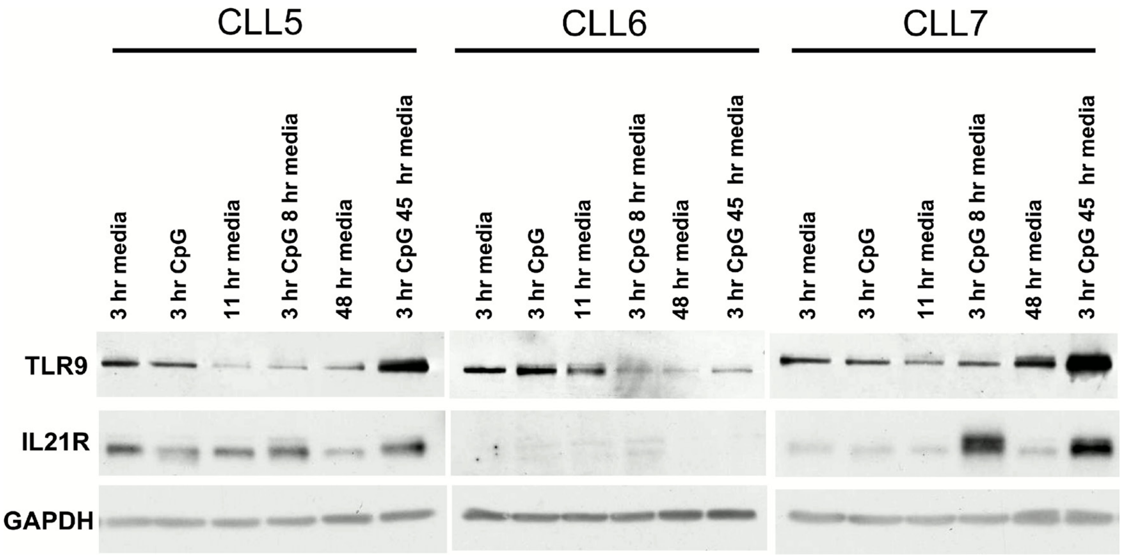

Supplementary Figure S2: TLR9 and IL21R protein in CLL patient samples. Immunoblot analysis of lysates from CLL B cells treated with CpG-685 for 3 hours, washed, then incubated in fresh media for a total time of 11 or 48 hours. Blots were probed for TLR9, IL21R, and GAPDH. Patient CLL6 corresponds to CLL6 in Figure 3c.

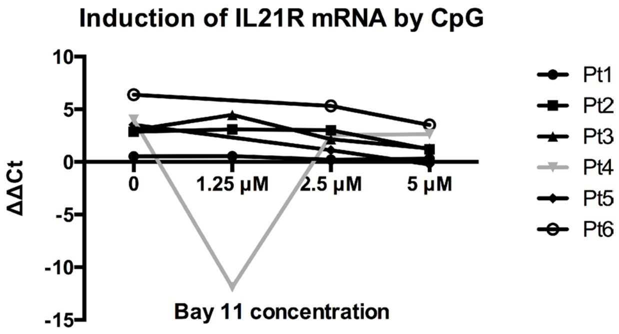

Supplementary Figure S3: Treatment with CLL B cells with Bay 11 diminishes CpG-685-mediated upregulation of IL21R. Real time RT-PCR indicates that 2.5 µM and 5 µM Bay 11 inhibits the transcriptional upregulation of IL21R by CpG-685. Data is shown as ddCt = media(IL21R-18S)-CpG(IL21R-18S). Pt4 is an influential outlier and was excluded from statistical analysis.
